# Core Temperature Responses in Elite Cricket Players during Australian Summer Conditions

**DOI:** 10.3390/sports6040164

**Published:** 2018-12-05

**Authors:** Sharon Stay, Michelle Cort, David Ward, Alex Kountouris, John Orchard, Justin Holland, Anna Saw

**Affiliations:** 1 Northside Sports Medicine, Stafford Heights, Queensland 4053, Australia; 2 Cricket Australia, Albion, Queensland 4010, Australia; michelle.cort@cricket.com.au; 3 Brisbane Northside Emergency, Chermside, Queensland 4032, Australia; david_ian_ward@hotmail.com; 4 Cricket Australia, Jolimont, Victoria 3002, Australia; alex.kountouris@cricket.com.au (A.K.); anna.saw@cricket.com.au (A.S.); 5 School of Public Health, University of Sydney, Camperdown, New South Wales 2006, Australia; john.orchard@cricket.com.au; 6 School of Exercise and Nutrition Sciences, Queensland University of Technology, Kelvin Grove, Queensland 4059, Australia; justin.holland@qut.edu.au

**Keywords:** core temperature, thermoregulation, cricket, exertional heat illness

## Abstract

This study aimed to observe core temperature responses in elite cricket players under match conditions during the summer in Australia. Thirty-eight Australian male cricketers ingested capsule temperature sensors during six four-day first-class matches between February 2016 and March 2017. Core temperature (Tc) was recorded during breaks in play. Batters showed an increase in Tc related to time spent batting of approximately 1 °C per two hours of play (*p* < 0.001). Increases in rate of perceived exertion (RPE) in batters correlated with smaller elevations in Tc (0.2 °C per one unit of elevation in RPE) (*p* < 0.001). Significant, but clinically trivial, increases in Tc of batters were found related to the day of play, wet bulb globe temperature (WBGT), air temperature, and humidity. A trivial increase in Tc (*p* < 0.001) was associated with time in the field and RPE when fielding. There was no association between Tc and WBGT, air temperature, humidity, or day of play in fielders. This study demonstrates that batters have greater rises in Tc than other cricket participants, and may have an increased risk of exertional heat illness, despite exposure to similar environmental conditions.

## 1. Introduction

The sport of cricket requires long hours of play during the hottest months of the year. Cricketers may spend up to eight hours on the field during a day’s play. Intermittent high intensity activity such as sprinting between wickets or chasing a ball to the boundary is required over many hours. Bowlers engage in short sprints and brief maximum exertion during delivery of the ball, up to 150 times per day. This intermittent exertion, together with exposure to environmental factors such as elevated air temperature, high summer humidity, and long periods of direct solar radiation may place cricketers at risk of exertional heat illness (EHI).

A four-year (2001–2004) epidemiological study of hospital records in New South Wales, Australia, identified eight cases of heat-related hospital presentations associated with recreational cricket participation [1]. These cases accounted for 7% of all heat-related presentations associated with sport and leisure activities, placing cricket amongst the top three sports to cause heat-related illness (alongside marathon running and golf). There has been one report of heat contributing to the death of a Namibian cricketer [2]. Documented incidents of heat illness in cricketers are likely to reflect extreme cases; less extreme presentations may be unreported or unrecognised, while still affecting individual athlete well-being and performance.

Tennis, similar to cricket, is a summer sport requiring intermittent high-intensity exertion. As a sporting organisation, Tennis Australia has implemented a heat policy to protect players, officials, and spectators [3]. Tennis has similar national participation rates to cricket (4.8% tennis versus 3.3% cricket) [4]. Many other sporting codes adopt policies relating to the prevention of EHI as a duty of care towards participating athletes and officials [5,6,7]. There is no formal heat policy for international cricket, which may reflect the lack of evidence and research to provide guidance. The International Cricket Council (ICC) states, “under conditions of extreme heat the umpires may permit extra intervals for drinks” [8]. In Australia, domestic cricket organisations provide guidelines for games in extreme heat, based upon umpire discretion and perceived environmental conditions.

This study was designed to observe the core temperature (Tc) responses of Australian first-class cricketers during typical summer playing conditions and determine if a relationship between core temperature and environmental conditions was present. Additionally, the variability of core temperature related to player position (batter, bowler, fielder, wicket keeper) under similar environmental conditions was to be determined.

## 2. Materials and Methods

### 2.1. Participants

Thirty-eight male cricketers from three state teams (Queensland, South Australia, and Western Australia) were involved in the study. All of the subjects gave their informed consent for inclusion before they participated in the study. Exclusion criteria included prior severe heat illness, febrile illness within 72 h of game commencement, prior reaction to CorTemp capsule, pre-existing systemic disease, inability to swallow capsules, and treatment with medications affecting ability to thermoregulate. The study was conducted in accordance with the Declaration of Helsinki, and the protocol was approved by the University of Queensland Medical Research Ethics Committee (Approval Number: 2015001242).

### 2.2. Design

This study was an observational cohort study, with data collected over four days of play during six first-class cricket games between February 2016 and February 2017. Games were held in Brisbane (February 2016, March 2016; latitude 27.4858° S), Adelaide (February 2016, February 2017; latitude 34.9155° S) and Perth (February 2016; latitude 31.9598° S).

### 2.3. Procedure

Participants ingested a CorTemp telemetry capsule (CorTemp, HQInc., Palmetto, FL, USA) approximately three hours prior to the start of play on each day of the scheduled four-day matches. Timing of ingestion was consistent with other studies that utilised ingestible telemetry devices [9].

Core temperature measurements were recorded using a hand-held CorTemp Data Monitor (CorTemp, HQInc., Palmetto, FL, USA) at seven set times during the day when athletes were accessible (pre-match, first session drinks break, lunch, second session drinks break, tea, third session drinks break, post-match). At each set time, athletes’ RPE (standard CR-10 Borg scale [10]), symptoms of heat illness, player position (batsman, bowler, fielder, wicket-keeper), ambient air temperature, humidity, and wet bulb globe temperature (WBGT) (WBGT Heat Stress Meter 800036, Sper Scientific Ltd., Scottsdale, AZ, USA) were recorded.

Standard fuelling and hydration practices were followed, and players were allowed free access to available food and fluids throughout the day. Daily first morning urinary specific gravity (USG) and body weight before play was recorded as per team protocol.

### 2.4. Statistical Analysis

Data was analysed using SPSS (Version 24.0. IBM Corp., Armonk, NY, USA), with confidence interval (CI) limits set at 90%, and significance set at *p* < 0.05. Extreme Tc readings that were inconsistent with clinical context were excluded from analysis (typically below 36 °C and above 41 °C, where visual inspection of the player and repeated readings demonstrated erroneous data), which is consistent with other studies utilising capsule telemetry [9].

Data was assessed for normality using visual inspection of frequency histograms and Shapiro–Wilk tests. As it was found that all of the variables were positively skewed (with the exception of Tc and humidity, which were negatively skewed), central tendency measures utilised the median and interquartile range (IQR). Generalised estimating equations were used to model the association between environmental (WBGT, air temperature, humidity) and match variables (day of play, time fielding or batting, RPE, player role) with the dependent variable Tc. Variables were first assessed by univariate analyses, and significant variables were subsequently combined in step-wise multivariate analyses. An exchangeable correlation structure, gamma distribution, and identity link function were used. To ascertain the certainty of a meaningful change, effects were calculated per two standard deviations of each independent variable, and probabilistic inferences were made according to the scale: 25–75%, possible; 75–95%, likely; 95–99.5%, very likely; >99.5%, most likely [11].

## 3. Results

Six hundred and ten valid Tc observations were recorded across the six four-day first-class matches; 47 observations were recorded during batting, 320 were recorded during fielding (bowler, fielder, wicket keeper), and 243 were recorded during off-field periods of play. Prior to the start of play on day one of each match, the mean Tc was 37.6 °C (90% CI 37.3–37.8). The average age of players was 26 years (19–37), mean body mass was 85.0 kg (83.2–86.8), and morning USG was 1.015 (1.011–1.020).

During batting, the duration of time batting and rate of perceived exertion (RPE) were most likely (>99.5%) associated with an increase in Tc (Table 1). Tc was observed to increase by 1.0 °C (0.6–1.4, *p* < 0.001) per session of batting (approximately two hours). Tc increased by 0.2 °C (0.2–0.3, *p* < 0.001) per unit increase in RPE. The combination of time batting and RPE was not observed to have a synergistic effect on Tc. Significant but clinically trivial increases in Tc were found related to WBGT, air temperature, humidity, and the day of play in batters.

During fielding, the duration of time in the field and RPE were associated with a likely (75–95%) but trivial increase in Tc (*p* < 0.001) (Table 2). This association held for all of the player roles in the field (bowler *p* < 0.001, wicket keeper *p* = 0.042, fielder *p* = 0.001). Analysis of this data demonstrated no association between Tc and WBGT, air temperature, humidity, or day of play in fielders.

## 4. Discussion

This study observed the core temperature responses of elite cricket players during first-class matches in Australian summer conditions. The most notable finding was an increase in Tc associated with time batting. Core temperature rose approximately 1 °C per two-hour session of batting. This temperature increase was not observed when fielding, suggesting that batting-specific factors affect normal thermoregulation. These factors could include: the accumulation of heat due to the intermittent high exertion of batting and running between wickets; maintaining a workload over a prolonged period of time; and reduced heat loss and retention of body heat by protective clothing and equipment worn by batsmen.

Protective equipment worn by batsmen may be a key factor contributing to elevated Tc. In addition to the uniform of long trousers and either short or long-sleeved shirt, batsmen may wear compression undergarments, protective padding of the lower legs, thigh, chest, and forearm, gloves, and a helmet. Body regions with high sweat rates during exercise include the forehead, chest, back, shoulders, forearms, and thighs [12]. Wearing protective clothing whilst playing cricket is likely to reduce heat dissipation [13]. The type of fabric worn may affect thermoregulation, but is not regulated by domestic cricket organisations. Material type was not recorded as part of this study. Interestingly, players wearing similar protective equipment (wicket keepers and close fielders) did not experience the same rise in Tc with time fielding. It is likely that, when wearing similar protective equipment, batting involves greater physical exertion than fielding positions that require protective equipment. This alone may be an important contributor to body heat accumulation.

The self-reported workload (RPE) of batters was also associated with an elevation of Tc. A five-point increase in RPE was associated with an increase of 1 °C. Day of play (sequentially day one to four) was not associated with Tc elevation, suggesting a negligible effect of cumulative thermal exposure, fatigue, or possible dehydration. Since domestic first-class matches are held over four days, heat acclimatisation over such a time period is unlikely to play a role in Tc elevation.

Environmental conditions such as air temperature and humidity are known to contribute to the risk of EHI [14]. This study found that environmental conditions (air temperature, WBGT, humidity) were only trivially associated with Tc. This may be due to acclimatisation in Australian cricketers, but there is no clear evidence to explain why this relationship was not more significant. The air temperatures recorded during this study were typical for an Australian summer, but were not extreme. Air temperatures recorded from these six matches ranged from a minimum of 19.6 °C to a maximum of 42.1 °C. Approximately one-third (35%) of temperatures recorded (48 of 137) were higher than 30 °C during the six matches studied.

Players were asked to report symptoms of EHI. Few symptoms were reported, but included dry mouth, abdominal cramping and post-match headache. These symptoms, when present, did not correlate with measured Tc elevation. There were 18 Tc measurements exceeding 39°; however, no player was diagnosed with EHI. There is wide interpersonal variability in tolerance to raised Tc [15]. Some athletes can reach Tc in excess of 41 °C without limitation of exercise performance or signs of heat illness [16].

In this study, individual player hydration was not regulated. Hydration is known to affect core temperature. In dehydrated athletes, Tc rises by 0.15–0.20 °C for each 1% of body mass lost during activity [16]. Significant fluid loss due to dehydration has been observed in match conditions in cricket [17], adding to the risk of EHI. It may be that elite cricket players, particularly fielders, are able to effectively thermoregulate using standard hydration and cooling strategies in environmental conditions similar to this study.

### 4.1. Limitations

Although CorTemp capsules were easy to use and well-tolerated by athletes, missed or erroneous readings limited the quantity and continuity of data, and hence the ability to draw conclusions. For example, intermittent extreme Tc readings (e.g., below 35 °C) were considered non-physiological, and were possibly due to delayed gastric transit and/or the ingestion of cold beverages.

Despite this limitation, ingestible telemetry devices have been shown to be a valid measure of Tc when compared to rectal temperature, both at rest and during periods of severe heat stress induced by exercise [18]. Researchers have noted a bias of less than 0.1 °C and a high level of agreement between intestinal and rectal Tc measurement using capsule devices [19]. Ingestible telemetry devices have been successfully utilised in a variety of competitive sports, including Australian rules football [20], soccer [21], cycling [22], triathlons [9], and motor racing [23].

In this study, no participant batted for longer than three hours. It is unlikely Tc would continue to increase at 1° per two-hour session, and unwise to extrapolate this data to prolonged batting spells. The self-regulation of exercise intensity and work rate would likely ensure that Tc did not rise excessively in the case of a long batting inning [16,24].

Our study found no correlation between reported symptoms of EHI and Tc. It is possible that under-reporting of symptoms may have occurred due to concerns regarding potential medical removal from the game.

Lastly, this study was limited to an elite cohort of cricketers in Australian summer conditions. To further advance the understanding of Tc responses in cricket, additional research is needed to observe longer batting stints, more extreme and diverse environmental conditions, female cricketers, and cricketers of different ages, ethnicities, and fitness levels.

### 4.2. Practical Applications

The data from this study suggest that in Australian cricketing conditions, batsmen may be at higher risk of Tc elevation than other players. Along with risks to player health, exercise in extreme heat is known to impair aerobic performance and reduce work rate due to the effects of thermal strain [25]. Cognitive function is also impaired with exposure to high temperatures [26].

In light of this, providing adequate cooling options for batsmen becomes more important. Potential cooling options include: the use of ice vests during breaks in play, cooled neck cloths, maintaining hydration with the ingestion of cooled beverages [25], temporary shade during breaks, longer drinks breaks, and extra drinks breaks in extreme conditions. Advances in sports clothing and helmet design may facilitate enhanced air circulation. A short duration cold water immersion has been shown to significantly lower Tc in the setting of ongoing submaximal exercise [27]. This may be applicable during longer breaks (lunch, tea) in first-class cricket. Pre-cooling, although useful in reducing Tc in sports lasting up to 40 minutes, is unlikely to be useful in cricket, due to its intermittent nature and long game duration [28].

Acclimatisation is one of the key individual factors in reducing the risk of EHI in athletes, and has been shown to improve athlete response to thermal stress [29,30]. Australian elite cricket players are generally well acclimatised, with many months of pre-season training, trial competition, and shorter format games before first-class matches begin. Care must be taken not to extrapolate these findings to athletes who have not been adequately acclimatised due to injury, geographical relocation, or late team inclusion, as they are more likely at risk of EHI.

With respect to player welfare, it is important to identify additional risk factors for EHI in individual cricket players. Intercurrent illness (viral or other), pre-existing disease, sunburn, recent concussion, prescription medications, illicit substance use, and alcohol intake can all increase heat storage by impairing thermoregulatory mechanisms [31]. If present in batsmen, these conditions could predispose them to significant increases in Tc, enhancing the risk of EHI.

## 5. Conclusions

The objective of this study was to observe Tc in elite cricket players during first-class matches in typical Australian summer conditions. Batters showed an increase in Tc related to time spent batting (1 °C per session) and RPE. Fielders did not experience the same elevation, despite exposure to the same environmental conditions. The observed elevations in Tc related to environmental conditions or day of play were small and likely to be clinically insignificant.

## Figures and Tables

**Table 1:** Median core temperature and environmental and match variables, and the modelled association between variables and core temperature when batting (*n* = 47).

Core Temperature and Independent Variables	Median (IQR)	B ^#^ (90% CI)	Std. Error	*p*
Core temperature (°C)	38.5 (37.7–39.3)	-	-	-
WBGT (°C)	23.7 (15.6–31.8)	0.024 (0.010–0.039)	0.0087	0.005 *
Temperature (°C)	27.6 (22.4–32.8)	0.027 (0.016–0.038)	0.0067	<0.001 **
Humidity (%)	52.7 (35.6–69.8)	0.013 (0.008–0.018)	0.0029	<0.001 **
Day of play	-	0.225 (0.076–0.373)	0.0903	0.013 *
Time batting (per playing day)	0.2 (0.0–0.4)	2.931 (1.805–4.057)	0.6846	<0.001 **
RPE	4 (2–6)	0.233 (0.193–0.272)	0.0242	<0.001 **

**^#^** B is rise in core temperature per unit increase in independent variable. * *p* < 0.05, ** *p* < 0.001. IQR = interquartile range, WBGT = wet bulb globe temperature, RPE = rating of perceived exertion (0–10).

**Table 2:** Median core temperature and environmental and match variables, and the modelled association between variables and core temperature when fielding (*n* = 320).

Core Temperature and Independent Variables	Median (IQR)	B ^#^ (90% CI)	Std. Error	*p*
Core temperature (°C)	38.0 (37.3–38.7)	-	-	-
WBGT (°C)	24.2 (17.0–31.4)	−0.001 (−0.014–0.012)	0.0079	0.902
Temperature (°C)	27.7 (20.9–34.5)	0.005 (−0.004–0.014)	0.0053	0.333
Humidity (%)	48.1 (34.9–61.3)	0.001 (−0.001–0.004)	0.0014	0.294
Day of play	-	−0.043 (−0.118–0.032)	0.0454	0.343
Time fielding (per playing day)	0.3 (0.0–0.8)	0.482 (0.324–0.640)	0.0961	<0.001 **
RPE	4 (2–6)	0.082 (0.058–0.106)	0.0144	<0.001 **
Time fielding: Bowler	-	0.524 (0.320–0.728)	0.1240	<0.001 **
Time fielding: Wicket keeper	-	0.559 (0.107–1.011)	0.2747	0.042 *
Time fielding: Fielder	-	0.421 (0.210–0.632)	0.1282	0.001 *

**^#^** B is rise in core temperature per unit increase in independent variable. * *p* < 0.05, ** *p* < 0.001. WBGT = wet bulb globe temperature, RPE = rating of perceived exertion (0–10).

## Supplementary Materials

Supplementary File 1
